# Association between vaginal washing and group B *Streptococcus* colonization from periconception through the first trimester of pregnancy in a cohort of Kenyan women

**DOI:** 10.1371/journal.pone.0344736

**Published:** 2026-03-18

**Authors:** Clayton S. Jisuvei, John Kinuthia, Barbra A. Richardson, Sujatha Srinivasan, Erica M. Lokken, Kishor Mandaliya, Walter Jaoko, Matthew Munch, Tina L. Fiedler, David N. Fredricks, Raymond Scott McClelland

**Affiliations:** 1 Department of Medical Microbiology and Immunology, University of Nairobi, Nairobi, Kenya; 2 Department of Obstetrics & Gynaecology, Kenyatta National Hospital, Nairobi, Kenya; 3 Research and Programs, Kenyatta National Hospital, Nairobi, Kenya; 4 Department of Global Health, University of Washington, Seattle, Washington, United States of America; 5 Department of Biostatistics, University of Washington, Seattle, Washington, United States of America; 6 Vaccine and Infectious Disease Division, Fred Hutchinson Cancer Center, Seattle, Washington, United States of America; 7 Pathcare Laboratory, Mombasa, Kenya; 8 Department of Epidemiology, University of Washington, Seattle, Washington, United States of America; 9 Department of Medicine, University of Washington, Seattle, Washington, United States of America; Universidade dos Açores Departamento de Biologia: Universidade dos Acores Departamento de Biologia, PORTUGAL

## Abstract

**Introduction:**

Vaginal washing has been associated with adverse reproductive health outcomes including pelvic inflammatory disease, reduced fecundability, and HIV acquisition. This analysis tested the hypothesis that vaginal washing is associated with increased risk of group B streptococcus (GBS) colonization.

**Method:**

Women planning pregnancies contributed monthly visits during which vaginal fluid specimens were collected and urine pregnancy testing was performed. In women who became pregnant, additional samples were collected at 9–12 weeks gestation. Broad-range 16S rRNA gene PCR with next generation sequencing (NGS) was performed to identify vaginal bacterial species. Generalized estimating equations with a log link, Poisson family, independent correlation structure and robust errors were used to generate prevalence ratios comparing the prevalence of GBS detection at vaginal washing visits versus non-vaginal washing visits.

**Results:**

The 189 women who became pregnant contributed 506 samples used in this analysis. Samples were collected at periconception 196 (38.9%), early first trimester 151 (29.8%), and late first trimester visits 159 (31.4%). The prevalence of GBS during the three time periods was 20/196 (10.2%), 11/151 (7.3%) and 2/159 (1.3%) respectively. Vaginal washing was practiced by 51/196 (26.0%), 27/151 (17.9%) and 32/159 (20.1%) participants during the three time periods. Compared to visits with no vaginal washing, there was no increased prevalence of GBS detection at visits where vaginal washing with water was reported (prevalence ratio [PR] 0.51, 95% confidence interval [CI] 0.16–1.62). However, the prevalence of GBS detection was nearly five-fold higher at visits when vaginal washing using water and soap was reported (PR 4.66, 95% CI 1.51, 14.33).

**Conclusion:**

Vaginal washing with soap and water was associated with a nearly five-fold increase in GBS prevalence. Future studies should evaluate this association in later pregnancy and peripartum. Cessation or modification of vaginal washing practices could be a useful strategy for decreasing GBS colonization.

## Introduction

Vaginal washing practices, defined as washing beyond the introitus using water, soap, or commercial products [1], can alter vaginal pH and shift the vaginal microbiota from *Lactobacillus*-dominated bacterial communities to microbiologically diverse vaginal bacterial communities composed of facultative and anaerobic species [2,3]. Vaginal washing practices have no known health benefits and have been associated with acquisition of bacterial vaginosis (BV), sexually transmitted infections (STIs), and HIV infection [3,4]. The proportion of women with vaginal group B streptococcus (GBS) colonization is higher in non-pregnant compared to pregnant women [4]. However, the incidence of invasive GBS disease is two times higher in women who are pregnant [5]. Vaginal GBS colonization in parturient women has been associated with second-trimester miscarriage, preterm birth, premature rupture of membranes, very-low-birth-weight delivery, and puerperal sepsis [4,6,7]. Exploratory studies investigating the prevalence and correlates of vaginal GBS colonization have identified low level of education, infection with *Candida albicans*, intermediate vaginal microbiota (Nugent score of 4–6), BV, recent vaginal intercourse, cervical ectopy, commercial sex work, and vaginal washing as correlates of vaginal GBS colonization in both pregnant and non-pregnant women [4,6,7].

Identifying modifiable risk factors associated with vaginal GBS colonization during pregnancy could lead to health recommendations aimed at reducing colonization as well as invasive disease. The analysis presented here builds on earlier exploratory studies of correlates of vaginal GBS colonization by testing the hypothesis that vaginal washing is associated with increased risk of GBS colonization during the periconception period and the first trimester of pregnancy.

## Methods

### Study design and population

Women enrolled in the Microbiota and Preterm Birth (MPTB) study, a prospective cohort study of HIV negative women planning pregnancies in Nairobi and Mombasa, Kenya, were eligible for inclusion in this exploratory analysis [8]. Eligibility for the parent study included age ≤ 45 years, HIV negative, trying to conceive, reported menses during the three months before the study or recent discontinuation of contraceptive methods associated with amenorrhea. Women were excluded from the MPTB study if they were pregnant at enrollment, had a history of a health condition associated with preterm birth, antibiotic use within four weeks before the study, or history of seeking care for infertility. The Kenyatta National Hospital – University of Nairobi Ethics and Research Committee and the University of Washington Human Subjects Research Committee approved the study. All participants provided written informed consent. Minors aged 14–17 years were included if emancipated under Kenyan law.

This analysis included data and samples from 189 women included in the parent study. Samples were collected between March 2019 and February 2020. The screening, enrollment, and exclusions resulting in this participant population have been published [9]. In brief, this analysis included women who became pregnant during the study and provided vaginal samples at two or more pregnancy time points.

### Study procedures

At enrollment, study staff conducted a structured face-to-face interview to collect data on participant demographics, sexual behavior, medical and reproductive history, and vaginal washing practices. To ascertain vaginal washing status, women were first asked, “Do you wash inside your vagina?” If women responded yes, a follow-up question was asked; “Do you wash at least the length of a fingertip beyond the vaginal introitus?” If needed, a pelvic model was used to illustrate. Women were considered to be performing vaginal washing if they answered ‘yes’ to both questions.

At the enrollment visit, study clinicians performed a speculum-assisted pelvic examination and collected five vaginal swabs. Each swab was rolled three times against the lateral vaginal wall, carefully avoiding overlapping areas to ensure sample quality. The first swab, which was collected using the Hologic Aptima collection kit, was used for STI testing. The next two samples were collected using push-off polyester swabs for vaginal microbiota analysis and placed in dry storage vials. A fourth sample was collected using a cotton-tipped swab to prepare a smear on a glass slide for BV Gram staining. The fifth sample, also collected with a cotton-tipped swab, was placed in a glass tube containing normal saline for wet mount microscopy. After collection, the glass slide was placed in a slide box, and the wet mount tubes were placed in a rack holder. Aptima and microbiota samples were placed in a transport flask containing ice. All samples were transported to the laboratory within three hours of collection for testing and long-term storage at −80°C.

If women were currently menstruating, enrollment visits were deferred until after menses. Laboratory confirmed STIs detected at enrollment and STI symptoms identified at any time during follow-up were treated per Kenya guidelines [10].

At monthly preconception visits, a urine pregnancy test was performed and information about sexual behavior, vaginal washing, and genitourinary symptoms in the prior four weeks was updated. During these visits, participants self-collected vaginal swabs after receiving a demonstration using a pelvic model. They were instructed to avoid collecting samples from overlapping areas of the vaginal walls to ensure sample quality. Swabs were handed to participants one at a time during the collection process. A total of four swabs were collected for molecular analysis of microbiota (2 swabs), vaginal Gram stain, and vaginal saline wet mount. Handling, transport, and storage of these swabs followed the same procedures as described for the enrollment swabs [11]. As with the enrollment visit, sample collection was deferred if women were currently menstruating.

Most participants were followed for up to 6 monthly preconception visits. Women who had discontinued depot medroxyprogesterone acetate (DMPA) contraception <6 months before enrollment were followed for up to 9 months of preconception time because of the delayed return to fertility after DMPA use. Women who had a positive urine pregnancy test self-collected vaginal swabs for storage at −80°C and were scheduled for a first-trimester ultrasound visit between 9–12 weeks of gestation. At the ultrasound visit, women also collected vaginal swabs as described for preconception visits, and study staff collected updated information on sexual behavior, vaginal washing, and medical history.

### Laboratory procedures

Enrollment vaginal samples were tested for *Neisseria gonorrhoeae, Chlamydia trachomatis,* and *Trichomonas vaginalis* (Aptima Combo-2 CT/NG Detection System and Aptima *Trichomonas vaginalis* assay*;* Hologic Inc., San Diego, CA). Vaginal Gram-stained slides were evaluated for BV using the method of Nugent and Hiller [12]. Vaginal yeast was detected on vaginal potassium hydroxide wet mount.

Vaginal GBS was detected using broad range 16S rRNA gene PCR with next-generation sequencing (NGS). Vaginal swabs stored at −80°C were thawed on ice and washed with 450 µL filtered 0.9% saline before pelleting for DNA extraction using the QIAamp BiOstic Bacteremia DNA Kit (Qiagen). DNA was eluted in 75 µL of EB buffer and 75 µL of filtered 0.2X Tris-EDTA buffer. PCR inhibition was measured using an internal amplification control quantitative PCR (qPCR) assay as previously described [13], and total bacterial load was estimated using a TaqMan based broad range qPCR assay targeting the 16S rRNA gene [14]. Amplicons from a broad range PCR targeting the V3-V4 region of the 16S rRNA gene were sequenced using the Miseq platform (Illumina) as previously described [15]. A mock community with known DNA concentrations of several vaginal bacteria was run as a positive control [15]. Air swabs from collection sites and extraction and sham swab controls were run to monitor for contamination during collection and processing. No-template water controls were run to monitor for potential PCR reagent contamination. The *DADA2* package was used for processing 16S rRNA gene sequence reads resulting in a list of unique sequence variants (SVs); taxonomy was assigned to the SVs by placing on a custom phylogenetic tree [15]. Sequences have been deposited to NCBI SRA (BioProject Accession: PRJNA1114047).

### Statistical analysis

The exposure variable for these analyses was vaginal washing, categorized as none, vaginal washing with water only, and vaginal washing with soap, with or without water. The primary outcome was detection of GBS using broad range 16S rRNA gene PCR with NGS. Bivariate analysis of the association between vaginal washing and GBS colonization was conducted using generalized estimate equations (GEE) with a log link, Poisson family, independent correlation structure, and robust errors. A multivariable analysis was used to adjust for potential confounding factors that were selected *a priori* based on their known or suspected associations with both vaginal washing and GBS colonization. Adjustment variables included age, level of education, frequency of condomless sex in the past month, presence of vaginal yeast on wet mount, and BV [4,6,7].

An additional exploratory analysis was conducted to examine the association between reproductive time points (periconception, early first trimester, late first trimester) and GBS detection. Bivariate analysis using GEE with a log link, Poisson family, independent correlation structure, and robust errors was conducted comparing GBS carriage in the periconception period (<28 days gestation) to the early (4 and 0/7–8 and 0/7 weeks) and late (8 and 1/7–13 and 6/7 weeks) first-trimester time points. A multivariable model was used to explore variables that could mediate the association between reproductive time points and GBS colonization, including vaginal washing, frequency of condomless sex in the past month, presence of vaginal yeast on wet mount, and BV.

## Results

The 189 participants included in this exploratory analysis contributed 506 total samples. One hundred and ninety-six (38.9%) samples were collected during the periconception period, 151 (29.8%) during early first trimester, and 159 (31.4%) during the late first trimester. Baseline characteristics of the study population are presented in Table 1. Over half of participants were in either the 25–29 (n = 62, 32.8%) or 30–34-year-old age group (n = 56, 29.6%). A majority had 12–15 years (n = 87, 46%) or over 16 years (n = 44, 22%) of formal education. Most had a monthly household income of between 10,000 and 40,000 KSh. (n = 77, 41.2%). Prior pregnancy was reported by 182 (96.3%) women. At enrollment, vaginal washing was practiced by 66 (34.9%) women, of whom 49 (74.2%) used water only and 17 (25.8%) used soap with water. None of the participants reported using other substances such as detergents or antiseptics for vaginal washing. At follow-up visits, vaginal washing in the past month was reported at 51/196 (26.0%) periconception, 27/151 (17.9%) early first-trimester, and 32/159 (20.1%) late first-trimester visits.

**Table 1:** Baseline characteristics of 189 Kenyan women planning a conception attempt.

Characteristic	N(%)
Demographic and Partnership	
Age	
<25	28 (14.8)
25-29	62 (32.8)
30-34	56 (29.6)
35-39	37 (19.6)
40-45	6 (3.2)
Education Level	
<8 years	8 (4.2)
8-11 years	52 (27.5)
12-15 years	87 (46.0)
≥16 years	42 (22.2)
Monthly Household Income (KSh)	
< 2,500	7 (3.7)
2,500 − 10,000	46 (24.3)
10,000-30,000	77 (40.7)
30,000-75,000	36 (19.0)
>75,000	21 (11.1)
Partner’s HIV-serostatus	
HIV-seronegative	138 (73.4)
HIV-seropositive	8 (4.3)
Unknown	42 (22.3)
Reproductive History	
Most recent contraceptive method	
None	50 (26.5)
Condoms only	7 (3.7)
OCP	4 (2.1)
DMPA	6 (3.2)
Copper IUD	60 (31.7)
Implant	61 (32.3)
Other	1 (0.5)
Ever Pregnant	182 (96.3)
Prior live birth	173 (91.5)
Among those with a prior pregnancy (n = 182):	
History of ectopic pregnancy	2 (1.1)
History of pregnancy loss (<20 weeks)	43 (23.6)
History of induced abortion	8 (4.4)
Number of menstrual cycles of prior conception attempt time	
No prior trying time	143 (75.7)
1-3 cycles	26 (13.8)
4-6 prior cycles	10 (5.3)
More than 6 cycles	7 (3.7)
Trying time unknown	3 (1.6)
Sexual behavior and vaginal washing in last 4 weeks	
Any vaginal washing	66
Among those with vaginal washing used (n = 66):	
Water only	49 (74.2)
Soap with water	17 (25.8)
Frequency of condomless sex in last 4 weeks at enrollment	
No sex	17 (9.1)
1-4	59 (31.2)
5-8	48 (25.4)
≥9	63 (33.3)
STI and BV	
History of PID	2 (1.1)
History of STI	3 (1.6)
*M. genitalium*	8 (4.2)
*T. vaginalis*	2 (1.1)
*C. trachomatis*	12 (6.3)
Yeast	13 (6.9)
BV (Nugent ≥7)	58 (30.7)

Abbreviation: BV-Bacterial vaginosis, IUD – Intrauterine device, KSh - Kenya shillings, OCP – Oral contraceptive pills, PID – Pelvic inflammatory disease, STI- Sexually transmitted infection,

Detection of GBS was observed at 25/396 (6.3%) visits with no vaginal washing, 3/93 (3.2%, PR 0.51, 95% CI 0.16–1.62) visits with vaginal washing using water alone, and 5/17 (29.4%, PR 4.66, 95% CI 1.51–14.33) visits with vaginal washing using soap and water. These associations were similar in analyses adjusted for age, education, frequency of condomless sex, presence of vaginal yeast, and presence of BV (Table 2).

**Table 2:** Association between vaginal washing and GBS colonization in a cohort of Kenyan women.

Type of Vaginal Washing	GBS Detected	Unadjusted		Adjusted^a^	
	N (%)	PR	95% CI	PR	95% CI
None	25/396 (6.3)	1	Referent	1	Referent
Water Only	3/96 (3.2)	0.51	0.16, 1.62	0.55	0.16, 1.88
Water & Soap	5/17 (29.4)	4.66	1.51, 14.33	5.11	1.93, 13.51

Abbreviation: CI, confidence interval; GBS, group B streptococcus; PR, prevalence ratio.

^a^Adjusted for age, education, frequency of condomless sex, presence of vaginal yeast, and bacterial vaginosis.

No vaginal washing was reported at 396 visits, vaginal washing with water alone was reported at 93 visits, and vaginal washing with soap and water was reported at 17 visits.

**Fig 1:**
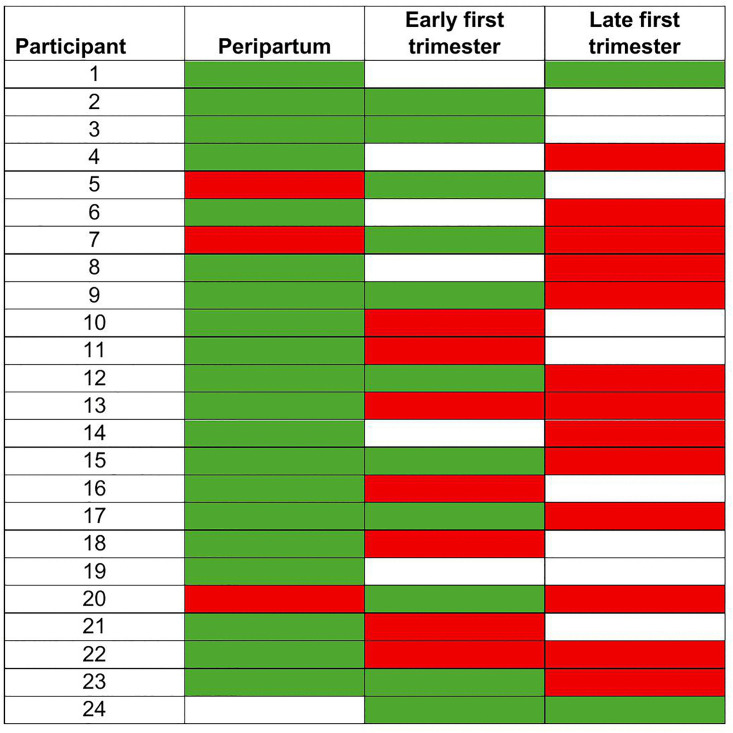
Group B *Streptococcus* detection at the peripartum, early first trimester, and late first trimester time points in 24 women with any group B *Streptococcus* detection. Detection of group B *Streptococcus* among study participants across three sampling time points: peripartum, early first trimester, and first trimester. Each row represents an individual participant. Color coding indicates GBS culture results as positive (green), negative (red), or sample not collected (white).

The prevalence of GBS colonization declined from periconception through late first-trimester, with GBS detection at 20/196 (10.2%) periconception visits, 11/151 (7.3%, PR 0.71, 95% CI 0.42–1.23) early first-trimester visits, and 2/159 (1.3%, PR 0.12, 95% CI 0.02–0.90) late first-trimester visits. Fig 1 illustrates the time points at which GBS was detected in the 24 women with GBS detection in one or more samples.

In multivariable analyses that included potential mediators including vaginal washing, frequency of condomless sex, and presence of vaginal yeast, results for both early first-trimester (adjusted PR [aPR] 0.80, 95% CI 0.44, 1.44) and late first-trimester samples (aPR 0.12, 95% CI 0.02, 0.89) were similar to the unadjusted analyses, suggesting that the relationship between reproductive time point and GBS colonization is not mediated through these variables. Because vaginal washing and BV could not be included in the same model due to sparse data, a separate multivariable model was constructed including BV, presence of vaginal yeast, and frequency of condomless sex. This model produced similar results (S1 Table).

## Discussion

This analysis of Kenyan women followed from periconception through the first trimester tested the hypothesis that vaginal washing is associated with increased risk for GBS colonization. The analysis demonstrated that vaginal washing with soap and water was associated with a nearly five-fold higher prevalence of vaginal GBS detection compared to no vaginal washing. In contrast, vaginal washing with water alone was not associated with increased GBS detection.

The results of these analyses mirror those of a cross-sectional study among nonpregnant and pregnant women at ≤14 weeks gestation, which reported that vaginal washing was associated with a more than two-fold increase in the odds of GBS colonization [4]. In contrast, a prospective study that sampled women at 22- and 33-weeks gestation reported no association between vaginal washing and GBS colonization [16]. Unlike the current analysis, these studies did not distinguish between substances used in vaginal washing. This paper adds to the evidence base by demonstrating that the association between vaginal washing and GBS colonization may be highly dependent on the substance used. In particular, vaginal washing with soap and water as opposed to washing with water alone may cause greater disruption to the cervicovaginal secretions, mucosa, and microbiota, setting up conditions that favor GBS colonization.

This study also identified a rapid decline in GBS colonization as pregnancies progressed from the periconception period through early first-trimester and late first-trimester. Prior studies have reported mixed findings on GBS colonization across pregnancies with some reporting a decline [17], while others have reported no change [16].

A major strength of this analysis was the differentiation of vaginal washing by type of substances used, which helped to identify increased GBS colonization with vaginal washing using soap with water, but not with water alone. This study also had several limitations. First, data used in this analysis were collected between the periconception period and late first trimester, which is not the most clinically important period for invasive GBS. Assessment of GBS in pregnancy is mainly performed during the third trimester due to its association with preterm birth, PROM, stillbirths, puerperal and neonatal sepsis [18]. Second, self-reporting of vaginal washing practices is subject to recall and social desirability bias. If vaginal washing, particularly vaginal washing with soap, was practiced by some women who reported no vaginal washing or washing with water only, this would likely lead to an under-estimation of the effect of this practice on GBS colonization. Third, because vaginal washing in the past month and GBS status were evaluated at the same visit, it is not possible to draw a firm causal link between vaginal washing and GBS colonization. Fourth, broad-range 16S rRNA gene PCR with NGS assay is less sensitive than taxon-directed PCR used by some studies [4], and could have missed detecting GBS below its detection threshold. Hence, the prevalence of GBS could be higher than that reported. Finally, the results of this analysis may not be generalizable to all populations. These results may be most generalizable to other African women in low- and middle-income countries.

In conclusion, the analysis presented in this paper tested the hypothesis that vaginal washing is associated with increased risk of GBS colonization during the periconception period and the first trimester of pregnancy. The findings demonstrate a strong association between recent vaginal washing using soap with water and vaginal GBS colonization. Future studies should examine the association between vaginal washing practices and GBS colonization in late pregnancy. Clinicians and public health professionals involved in women’s health should provide a clear message that vaginal washing has no known health benefits and may be associated with adverse sexual and reproductive health outcomes.

## Supporting information

S1 Table Multivariable relationship between GBS and vaginal washing. This multivariable analysis utilized a generalized estimating equation model with a binomial family, log link, and independent correlation structure. Potential confounding variables included in the model were age, education, frequency of condomless sex, presence of vaginal yeast, and presence of BV. Abbreviation: aPR, adjusted prevalence ratio; CI, confidence interval.
(DOCX)

## References

[1] Martin Hilber A, Hull TH, Preston-Whyte E, Bagnol B, Smit J, Wacharasin C, et al. A cross cultural study of vaginal practices and sexuality: implications for sexual health. Soc Sci Med. 2010;70(3):392–400. doi: 10.1016/j.socscimed.2009.10.023 19906477

[2] Linhares IM, Summers PR, Larsen B, Giraldo PC, Witkin SS. Contemporary perspectives on vaginal pH and lactobacilli. Am J Obstet Gynecol. 2011;204(2):120.e1-5. doi: 10.1016/j.ajog.2010.07.010 20832044

[3] Low N, Chersich MF, Schmidlin K, Egger M, Francis SC, van de Wijgert JHHM, et al. Intravaginal practices, bacterial vaginosis, and HIV infection in women: individual participant data meta-analysis. PLoS Med. 2011;8(2):e1000416. doi: 10.1371/journal.pmed.1000416 21358808 PMC3039685

[4] Cools P, Jespers V, Hardy L, Crucitti T, Delany-Moretlwe S, Mwaura M, et al. A Multi-Country Cross-Sectional Study of Vaginal Carriage of Group B Streptococci (GBS) and Escherichia coli in Resource-Poor Settings: Prevalences and Risk Factors. PLoS One. 2016;11(1):e0148052. doi: 10.1371/journal.pone.0148052 26811897 PMC4727807

[5] Deutscher M, Lewis M, Zell ER, Taylor TH Jr, Van Beneden C, Schrag S, et al. Incidence and severity of invasive Streptococcus pneumoniae, group A Streptococcus, and group B Streptococcus infections among pregnant and postpartum women. Clin Infect Dis. 2011;53(2):114–23. doi: 10.1093/cid/cir325 21690617

[6] Rocchetti TT, Marconi C, Rall VLM, Borges VTM, Corrente JE, da Silva MG. Group B streptococci colonization in pregnant women: risk factors and evaluation of the vaginal flora. Arch Gynecol Obstet. 2011;283(4):717–21. doi: 10.1007/s00404-010-1439-8 20349243

[7] Cho C-Y, Tang Y-H, Chen Y-H, Wang S-Y, Yang Y-H, Wang T-H, et al. Group B Streptococcal infection in neonates and colonization in pregnant women: An epidemiological retrospective analysis. J Microbiol Immunol Infect. 2019;52(2):265–72. doi: 10.1016/j.jmii.2017.08.004 28882582

[8] Lokken EM, Mandaliya K, Srinivasan S, Richardson BA, Kinuthia J, Lannon S, et al. Impact of preconception vaginal microbiota on women’s risk of spontaneous preterm birth: protocol for a prospective case-cohort study. BMJ Open. 2020;10(2):e035186. doi: 10.1136/bmjopen-2019-035186 32102825 PMC7045118

[9] McClelland RS, Lokken EM, Kinuthia J, Srinivasan S, Richardson BA, Jaoko W, et al. A prospective cohort study examining the association between the periconceptual vaginal microbiota and first-trimester miscarriage in Kenyan women. Paediatr Perinat Epidemiol. 2024;38(7):599–611. doi: 10.1111/ppe.13099 38949435 PMC11590749

[10] National AIDS & STI Programme of Kenya. Kenya National Guidelines for Prevention, Management, and Control of Sexually Transmitted Infections. Nairobi, Kenya2018.

[11] Bai G, Gajer P, Nandy M, Ma B, Yang H, Sakamoto J, et al. Comparison of storage conditions for human vaginal microbiome studies. PLoS One. 2012;7(5):e36934. doi: 10.1371/journal.pone.0036934 22655031 PMC3360033

[12] Nugent RP, Krohn MA, Hillier SL. Reliability of diagnosing bacterial vaginosis is improved by a standardized method of gram stain interpretation. J Clin Microbiol. 1991;29(2):297–301. doi: 10.1128/jcm.29.2.297-301.1991 1706728 PMC269757

[13] Khot PD, Ko DL, Hackman RC, Fredricks DN. Development and optimization of quantitative PCR for the diagnosis of invasive aspergillosis with bronchoalveolar lavage fluid. BMC Infect Dis. 2008;8:73. doi: 10.1186/1471-2334-8-73 18510764 PMC2440748

[14] Srinivasan S, Hoffman NG, Morgan MT, Matsen FA, Fiedler TL, Hall RW, et al. Bacterial communities in women with bacterial vaginosis: high resolution phylogenetic analyses reveal relationships of microbiota to clinical criteria. PLoS One. 2012;7(6):e37818. doi: 10.1371/journal.pone.0037818 22719852 PMC3377712

[15] Srinivasan S, Chambers LC, Tapia KA, Hoffman NG, Munch MM, Morgan JL, et al. Urethral Microbiota in Men: Association of Haemophilus influenzae and Mycoplasma penetrans With Nongonococcal Urethritis. Clin Infect Dis. 2021;73(7):e1684–93. doi: 10.1093/cid/ciaa1123 32750107 PMC8492123

[16] Furfaro LL, Nathan EA, Chang BJ, Payne MS. Group B streptococcus prevalence, serotype distribution and colonization dynamics in Western Australian pregnant women. J Med Microbiol. 2019;68(5):728–40. doi: 10.1099/jmm.0.000980 31013212

[17] Kwatra G, Adrian PV, Shiri T, Buchmann EJ, Cutland CL, Madhi SA. Serotype-specific acquisition and loss of group B streptococcus recto-vaginal colonization in late pregnancy. PLoS One. 2014;9(6):e98778. doi: 10.1371/journal.pone.0098778 24979575 PMC4076185

[18] ACOG. Prevention of group B streptococcal early-onset disease in newborns. Obstet Gynecol 2020:51–72. 10.1097/AOG.0000000000003668 31977795

