# Supplementary material for: Association between vaginal washing and group B Streptococcus colonization from periconception through the first trimester of pregnancy in a cohort of Kenyan women
Source: PLoS One. 2026 Mar 18;21(3):e0344736. doi: 10.1371/journal.pone.0344736 (PMC12998844; doi:10.1371/journal.pone.0344736)
Supplement: S1 Table — This multivariable analysis utilized a generalized estimating equation model with a binomial family, log link, and independent correlation structure. Potential confounding variables included in the model were age, education, frequency of condomless sex, presence of vaginal yeast, and presence of BV. Abbreviation: aPR, adjusted prevalence ratio; CI, confidence interval. (DOCX) [file pone.0344736.s001.docx]

**S1 Table**

|  |  |  |
| --- | --- | --- |
|  |  |  |

| **Type of Vaginal Washing** | **aPR (95% CI)** | **p-value** |
| --- | --- | --- |
| **None** | 1.0 | **Referent** |
| **Water Only** | 0.56 (0.16, 1.89) | **0.3** |
| **Water & Soap** | 5.11 (1.92, 13.61) | **0.001** |
